# Modelling and simulation of brinicle formation

**DOI:** 10.1098/rsos.230268

**Published:** 2023-10-25

**Authors:** Felipe Gómez-Lozada, Carlos Andrés del Valle, Julián David Jiménez-Paz, Boyan S. Lazarov, Juan Galvis

**Affiliations:** ^1^ Departamento de Física, Universidad Nacional de Colombia, Carrera 45 No. 26-85, Edificio Uriel Gutiérrez, Bogotá D.C., Colombia; ^2^ Departamento de Matemáticas, Universidad Nacional de Colombia, Carrera 45 No. 26-85, Edificio Uriel Gutiérrez, Bogotá D.C., Colombia; ^3^ Lawrence Livermore National Laboratory, Livermore, CA 94550, US

**Keywords:** finite-element method, nonlinear dynamics, multiphysics, phase change, ocean dynamics, chemical garden

## Abstract

Below the Arctic sea ice, under the right conditions, a flux of icy brine flows down into the sea. The icy brine has a much lower fusion point and is denser than normal seawater. As a result, it sinks while freezing everything around it, forming an ice channel called a brinicle (also known as ice stalactite). In this paper, we develop a mathematical model for this phenomenon, assuming cylindrical symmetry. The fluid is considered to be viscous and quasi-stationary. The heat and salt transport are weakly coupled to the fluid motion and are modelled with the corresponding conservation equations, accounting for diffusive and convective effects. Finite-element discretization is employed to solve the coupled system of partial differential equations. We find that the model can capture the general behaviour of the physical system and generate brinicle-like structures while also recovering dendrite composition, which is a physically expected feature aligned with previous experimental results. This represents, to our knowledge, the first complete model proposed that captures the global structure of the physical phenomenon even though it has some discrepancies, such as brine accumulation.

## 1. Introduction

**Figure 1.**
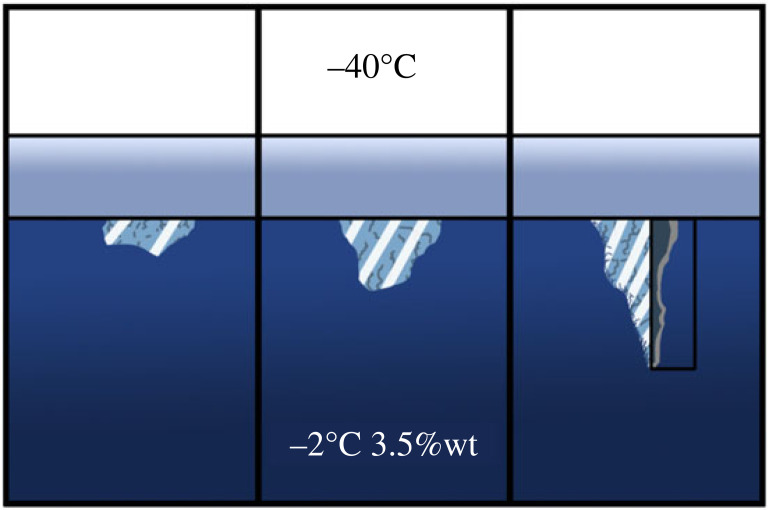
Diagram of a brinicle formation, the outside air (white) is at $-40^{\circ }C$ while the ocean (blue) is at $-2^{\circ }C$ and at a salinity of 3.5% wt. From the thick layer of ice (grey), a hollow chuck of ice, called a brinicle, grows downwards because of the cold brine flow inside it. The figure is inspired by [1,4].

Brinicles are naturally occurring inverse chemical gardens in the form of ice channels that freeze owing to a low-temperature brine flux from the surface ice [1]. They usually grow from a few centimetres to a metre and only appear in winter in the polar regions. During this season, the temperature above the ice drops from $-10^{\circ }C$ to $-40^{\circ }C$, while below the ice, the temperature remains at $-2^{\circ }C$ [1]. Hence, a temperature gradient appears going from the sea to the atmosphere. As the seawater loses heat, ice crystals nucleate beneath the ice. These crystals arrange themselves in horizontal bi-dimensional sheets called platelets [2]. Then, these platelets start to float, trapping seawater between layers of ice [3]. Part of the trapped seawater incorporates into the ice leaving behind the salt. As a result, the remaining seawater becomes extremely cold and salty forming cold brine. Eventually, the brine flows down the cracks of the ice into the sea. Seawater is nearly at its fusion point, and the denser brine sinks, freezing the water around it and forming a stalactite of ice called a brinicle. A diagram of a brinicle can be seen in figure 1.

There is a good amount of research on brinicles and related phenomena. For example, these forms are often studied to determine whether some ice-covered worlds, such as Jupiter’s moon Europa, are suitable for life [5,6]. The work [7] shows experimental studies analysing the brinicle formation process in a controlled laboratory environment. Furthermore, the effects of brine rejection and salt redistribution on polar regions, for example, anchor ice, can be found in [8]. A model for a similar physical phenomenon, the so-called black smoker, is shown in [9]. However, a complete model together with a numerical study of brinicle formation has yet to be proposed. Hence, the present work represents a first step into the simulation of these types of systems, which is also relevant to a wide range of applications resembling closely the partial differential equations (PDEs) used for a brinicle formation. Among others [10], these include phase-changing liquids in industrial processes [11], molten metals [12], thermal energy storage [13–16], smart textiles [17], and data storage [18,19]. Furthermore, the brinicle formation phenomenon also plays an important role in climate models [20].

In this article, we propose a model, described by a system of PDEs, to describe the formation of a brinicle in the Arctic sea. We consider a three-dimensional case with angular symmetry, which allows us to reduce the computational model to a two-dimensional computational domain. The decrease in computational cost enables us to account for different physical aspects, such as heat transfer, water flow and salt transport, with phase change included in each field. For the fluid motion, we used the stream function-vorticity formulation in the Stokes equation, including the Brinkman penalization [21,22] that zeros velocity in solid regions. The latter is computationally cheaper and much easier to implement than the boundary tracking method, which is usually used in some industrial-oriented numerical methods for simulating phase-changing fluids [23]. Moreover, we consider properties like phase-dependent diffusion while other parameters like viscosity are kept constant. We used the enthalpy formulation [24,25] to account for the energy required to perform the phase change, which includes the latent heat as an effective heat capacity. The numerical discretization is performed with the Galerkin finite-element method (FEM) [26] for the spatial discretization of the coupled PDE describing the physics of the problem. The evolution of the state fields is obtained with an implicit fourth-order Runge–Kutta method with an adaptive time step [27]. The numerical studies were performed with the help of the MFEM (https://mfem.org/) finite-element discretization library [28,29].

## 2. Physical problem

**Figure 2.**
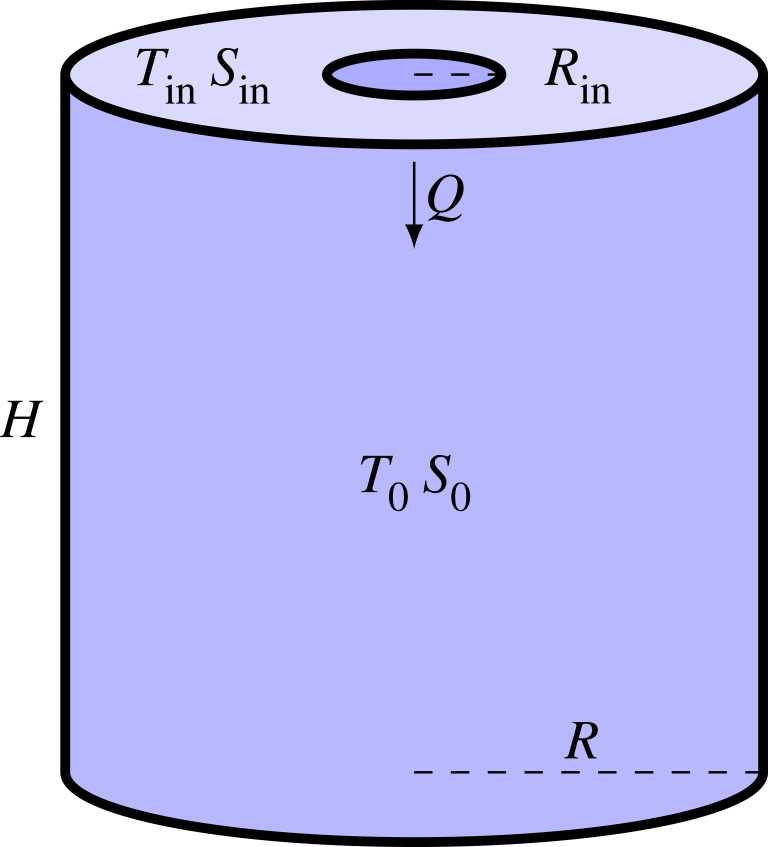
The domain of the study corresponds to a cylinder of radius *R* and height *H*. The cylinder is filled with liquid seawater (*T*_0_, *S*_0_). Cold brine with, temperature *T*_in_ and salinity *S*_in_, enters from the inlet of radius *R*_in_, located at the top centre of the domain. *Q* denotes the brine flux.

Our study is focussed on an experimental setting created by S. Martin and reported in [7], where the ice formation is reproduced by injecting cold brine through a hose into a cylindrical tank full of seawater. Following the experiment closely, we run the simulations in a cylindrical domain as shown in figure 2 with radius *R* and height *H* that is initially filled with seawater at temperature *T*_0_ and salinity *S*_0_. The cylinder axis goes through the middle of a circular inlet with radius *R*_in_ from where a flux *Q* of brine with temperature *T*_in_ and salinity *S*_in_ enters the tank. The bottom of the domain is assumed as an outlet that is located far from the inlet. The rest of the cylinder walls are assumed as insulated boundaries.

Our goal is to study the formation of the ice structure. Depending on the temperature, at every time instance, part of the domain is occupied with fluid and the other part with solid. The domain is occupied with a fluid phase for all points, where *T* ≥ *T*_*f*_, where the fusion temperature *T*_*f*_ is a function of salinity. The temperature and the salinity are time-dependent fields with evolution depending mainly on diffusive and convective effects and the latent heat in the energy transport. Furthermore, the diffusivity rates are different in each phase with functional forms and numerical values shown in detail in appendix A.

To model the phase change, we consider the domain like a porous medium where the permeability lets the fluid flow freely in the liquid regions and slows it down in the solid parts. Moreover, the liquid part of the domain is considered viscous and incompressible, where we include a buoyant force to account for movement caused by density differences produced by temperature and salinity gradients. As the phase change process usually occurs at long intervals, we assume the flow to be steady, neglecting the transient effects. Therefore, we calculate the steady-state velocity field ***V*** for a given set of temperature and salinity.

We aim to simplify the problem and propose a model in which all variables are dimensionless by scaling each quantity and equation with the corresponding reference factors. Specifically, for length and time, the scale factors are *R*_in_ and $2\pi R_{\mathrm{in}}^{3}/Q$, respectively. The main reason is that the inlet flux and the radius govern the characteristic time and length behaviour of the model. The time scale is selected in such a way that *Q*/2*π* is equal to one. The latter simplifies the boundary conditions of the flow equations. Regarding the temperature and salinity, we consider the following linear mapping:

2.1 $$f\;(X)=\frac{X_{\mathrm{in}}-X}{X_{\mathrm{in}}-X_{0}},$$

where *X* represents either the temperature or salinity. The idea is to apply the above function to each field instead of a reference factor to set the inflow conditions to zero, simplifying the numerical analysis. Moreover, the initial conditions are set to one, and a scale factor for both variables is given by |*X*_in_ − *X*_0_|.

## 3. Governing equations

Our computational domain is defined as Ω=[0,R]×[0,2π]×[0,H], and the state equation are defined in cylindrical coordinates (r,θ,z)∈Ω. The physical problem has angular symmetry allowing the angular coordinate *θ* to be removed from the equations. Thus, the original problem is defined entirely in the two-dimensional domain given by $\Omega^{\prime }=[0,R]\times [0,H]$ with coordinates $(r,z)\in \Omega^{\prime }$.

Inside the two-dimensional computational domain, we consider the usual gradient, divergence and curl operators, modified to account for the angular symmetry as

3.1 $$\nabla^{\prime }f\;(r,z)=\frac{\partial f}{\partial r}{\hat{e}}_{r}+\frac{\partial f}{\partial z}{\hat{e}}_{z}$$

and

3.2 $$\nabla^{\prime }\cdot f(r,z)=\frac{\partial f_{r}}{\partial r}+\frac{\partial f_{z}}{\partial z},$$

where ${\hat{e}}_{r}$ and ${\hat{e}}_{z}$ are unit vectors. The operators in the *r* − *z* plane are defined as

3.3 $$\nabla f\;(r,z)=\nabla^{\prime }f,$$

3.4 $$\nabla \cdot f(r,z)=\frac{1}{r}\nabla^{\prime }\cdot (rf)$$

3.5 $$\mathrm{and}\;\nabla \times f(r,z)=\frac{1}{r}R_{\frac{\pi }{2}}\nabla^{\prime }(rf_{\theta })+\nabla^{\prime }\cdot \left(R_{\frac{\pi }{2}}\left(\begin{array}{c} \;f_{r}\\ f_{z} \end{array}\right)\right){\hat{e}}_{\theta },$$

where $R_{\pi /2}=\left(\begin{array}{cc} 0 & -1\\ 1 & 0 \end{array}\right)$ is a rotation matrix by $90^{\circ }$, ${\hat{e}}_{i}$ are unit vectors in their corresponding directions, e.g. the angular direction, and $f_{\theta }=f\cdot {\hat{e}}_{\theta }$. With the help of equation (3.5), an identity regarding a double curl operator can be written as

3.6 $$\nabla \times (g(r,z)\nabla \times (f\;(r,z){\hat{e}}_{\theta }))=-\nabla^{\prime }\cdot \left(\frac{g}{r}\nabla^{\prime }(rf)\right){\hat{e}}_{\theta },$$

which will be used later for further simplifications. In the following subsections, we define the equations for thermal transport, salt transport and fluid motion in the two-dimensional domain with the corresponding boundary conditions.

### 3.1. Heat and salt transport

According to §2, for the heat and salinity transport, we consider diffusive and convective effects, which can be modelled with the following equation [30]:

3.7 $$\lambda_{1}\left(\frac{\partial \phi }{\partial t}+V\cdot \nabla \phi \right)-\nabla \cdot (\lambda_{2}\nabla \phi )=0,$$

where *ϕ* is the temperature or salinity field, ***V*** is the velocity field. The coefficients *λ*_1_ and *λ*_2_ correspond to the volumetric heat capacity and thermal conductivity in the heat problem. For the salinity transport, these coefficients correspond to unity and mass diffusivity.

Multiplying equation equation (3.7) with the Jacobian *r* and using the pseudo-cartesian operators from equations (3.3) and (3.4) results in

3.8 $$r\lambda_{1}\left(\frac{\partial \phi }{\partial t}+V\cdot \nabla^{\prime }\phi \right)-\nabla^{\prime }\cdot (r\lambda_{2}\nabla^{\prime }\phi )=0,$$

which defines the governing transport equation model in the cylindrical coordinate system.

Written explicitly the transport equations for the temperature and salinity are given as

3.9 $$r\left(1+\frac{1}{Ste}\delta (T-T_{f})\right)\left(\frac{\partial T}{\partial t}+V\cdot \nabla^{\prime }T\right)-\nabla^{\prime }\cdot \left(\frac{r}{Pe_{T}}\nabla^{\prime }T\right)=0$$

and

3.10 $$r\left(\frac{\partial S}{\partial t}+V\cdot \nabla^{\prime }S\right)-\nabla^{\prime }\cdot \left(\frac{r}{Pe_{S}}\nabla^{\prime }S\right)=0,$$

where *Pe*_*T*_ and *Pe*_*S*_ are the Péclet numbers that refer to the ratios of diffusion and convective rates for each equation [31] with the following functional form:

3.11 $$Pe(T,S)=\left\{ \begin{array}{cc} Pe_{\mathrm{liquid}}\; & T>T_{f}\;(S),\;\\ Pe_{\mathrm{solid}}\; & T<T_{f}\;(S),\; \end{array}\right.$$

where *Pe*_liquid_ and *Pe*_solid_ are the constant Péclet numbers from each phase.

The constant *Ste* is called the Stefan number and represents the ratio of the sensible and latent heat [23]. It is important to denote that the contribution in the form of a Dirac delta function in equation (3.9) results from the discontinuity on the enthalpy across the phase transition characterized by the fusion temperature *T*_*f*_. The term is associated with the energy needed to complete this process, namely the latent heat. Including it directly in the heat equation is often referred to as the enthalpy method [32]. The discussion regarding the construction of the Stefan number and each Péclet number with respect to the physical parameters and their corresponding values can be found in appendix A. It can be seen from the coefficients of equations (3.9) and (3.10) that these are highly nonlinear, which represents the principal difficulty of their solution.

### 3.2. Flow equations

Following the discussion in §2, the velocity field used in the transport equations for salinity and temperature is modelled with the help of the Stokes equations [33]:

3.12 ∇⋅V=0

and

3.13 $$\frac{1}{Da}V-\nabla^{2}V+\nabla p=-\frac{Ar}{Re}{\hat{e}}_{z},$$

where *p* is the pressure, *Re* is the constant Reynolds number representing the ratio between inertial and viscous forces, and *Ar* is the Archimedes number which is the ratio between buoyant forces and viscosity stresses [31]. The last number is proportional to density differences, i.e. it is a function of both temperature and salinity and is specified in appendix A, along with the construction and the value of the Reynolds number.

In addition, we include a Brinkman penalization [21] associated with the Darcy number *Da* , which in the limit is given by

3.14 $$Da(T,S)=\left\{ \begin{array}{cc} \infty \; & T>T_{f}\;(S),\;\\ 0\; & T<T_{f}\;(S).\; \end{array}\right.$$

The penalization is large, ideally approaching infinity, in the solid regions and is zero in liquid ones. However, numerical evaluation of the penalization requires that the Darcy number is strictly larger than zero. Therefore, *Da* is chosen to be very small to prevent flow in the solid regions and, at the same time, sufficiently large to avoid issues with the discretization and the numerical solution of the resulting algebraic equations. In connection to the above penalization model, we want to point out that in an actual physical scenario, any solid ice formed during solidification can be immobile only if connected to the upper ice sheet. The rest of the ice floats freely and is advected by the fluid. On the other hand, the proposed model does not allow the formed ice to move freely with the flow. The above limitation may result in non-physical fluid blockage during the brinicle growth process, discussed in detail in the results section.

To simplify the equation, we define stream ***ψ*** and the vorticity functions ***ω*** as follows:

3.15 V=∇×ψandω=∇×V.

Substituting them into the Stokes equations and taking the curl results in the following system:

3.16 ω−∇×∇×ψ=0

and

3.17 $$\nabla \times \nabla \times \omega +\nabla \times \left(\frac{1}{Da}\nabla \times \psi \right)=\nabla \times \left(-\frac{Ar}{Re}{\hat{e}}_{z}\right).$$

Owing to the axial symmetry of the problem, the stream and the vorticity have non-zero components only in the ${\hat{e}}_{\theta }$ direction. Thus we can model them with scalar fields *ψ* and *ω* as follows:

3.18 $$\psi =-\frac{\psi }{r}{\hat{e}}_{\theta }\;\mathrm{and}\;\omega =\frac{\omega }{r}{\hat{e}}_{\theta }.$$

Instead of solving for a two-dimensional velocity field ***V*** and a scalar pressure field *p*, we can solve for two scalar fields, *ψ* and *ω*, reducing significantly the computational cost.

Equations (3.16) and (3.17) are simplified to

3.19 $$\omega -r\nabla^{\prime }\cdot \left(\frac{1}{r}\nabla^{\prime }\psi \right)=0$$

and

3.20 $$-r\nabla^{\prime }\cdot \left(\frac{1}{r}\nabla^{\prime }\omega \right)+r\nabla^{\prime }\cdot \left(\frac{1}{Da}\frac{1}{r}\nabla^{\prime }\psi \right)=\frac{r}{Re}\frac{\partial Ar}{\partial r}.$$

It is important to denote that the symmetric pattern of these equations is a consequence of the addition of a negative sign in the stream function equation (3.18). Solving equations (3.19) and (3.20) allows us to obtain both fields *ω* and *ψ*, and the velocity field ***V*** is calculated as

3.21 $$V=-\frac{1}{r}R_{\frac{\pi }{2}}\nabla^{\prime }\psi .$$

### 3.3. Initial and boundary conditions

**Figure 3.**
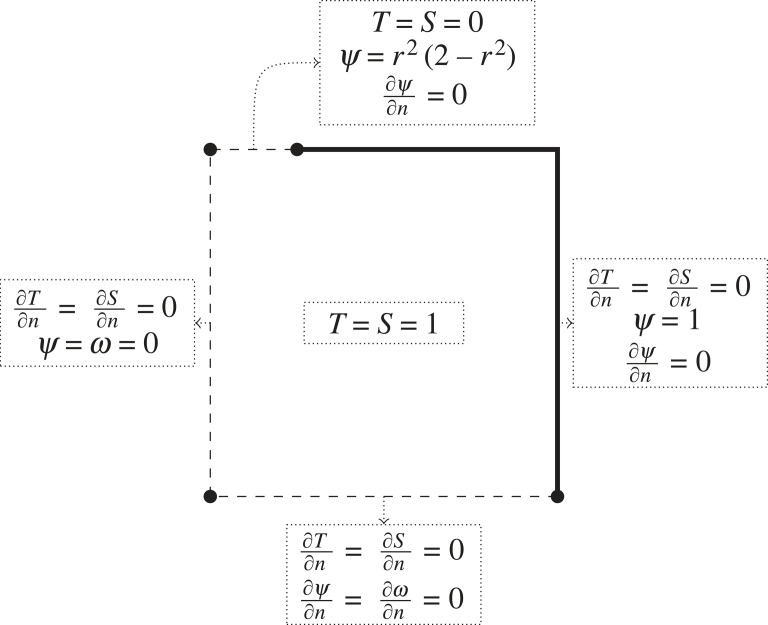
Initial and boundary conditions after simplification of the model owing to symmetry for the principal evolution equations. Centre: initial conditions; top: inlet boundary; left: symmetric boundary; right: closed boundary; bottom: outlet boundary. The parameters are set to simulate Martin’s [7] experimental set-up.

Equations (3.9) and (3.10) require the definition of initial and boundary conditions. On the other hand, equations (3.19) and (3.20) are non-time-dependent and only require boundary conditions. Based on the discussion in the beginning of §2, we use the following boundary and initial conditions (figure 3):

— *initial conditions:* constant value for temperature and salinity on liquid seawater values (*T*_0_, *S*_0_), which is (1, 1) in dimensionless form;
— *symmetric boundary:* this boundary corresponds to the centre of the cylinder, and there can be no radial transport since the direction is not defined. Thus, we set a zero Neumann condition on both temperature and salinity. As the stream function and vorticity are the angular components of a vector, the same argument applies, therefore both of them have a zero Dirichlet condition on this boundary;
— *inlet boundary:* the temperature and salinity have the value of the inflow brine (*T*_in_, *S*_in_). For the velocity field, we assume a vertical Poiseuille flow [33] typical in cylindrical pipes:
  3.22 $$V_{\mathrm{in}}=-\frac{2Q}{\pi R_{\mathrm{in}}^{2}}\left(1-{\left(\frac{r}{R_{\mathrm{in}}}\right)}^{2}\right){\hat{e}}_{z}.$$
  Based on the velocity profile and integrating equation (3.21) while preserving continuity on the stream function with the symmetric boundary, we derive a Dirichlet condition for the stream function given as
  3.23 $$\psi =\frac{Q}{2\pi }{\left(\frac{r}{R_{\mathrm{in}}}\right)}^{2}\left(2-{\left(\frac{r}{R_{\mathrm{in}}}\right)}^{2}\right).$$
  To enforce a null tangential velocity, we also apply a zero Neumann boundary to the stream function according to equation (3.21). In the dimensionless form, the Dirichlet conditions transform to (0, 0) for temperature and salinity and *r*^2^(2 − *r*^2^) for the stream function;
— *closed boundary:* there can be no transport in the normal direction of the boundary, therefore a zero Neumann condition for temperature and salinity is set. On the other hand, zero Neumann and constant Dirichlet conditions for the stream function enforce that the normal and tangent velocities are zero according to equation (3.21). To preserve the stream function continuity, the Dirichlet boundary is set to *Q*/2*π*, which equals one in dimensionless form; and
— *outlet boundary:* we assume far-field conditions where all gradients normal to the boundary are set to zero. In the case of the stream function, this enforces a zero tangent velocity.

## 4. Finite-element discretization

We used an unstructured mesh with triangular elements for discretizing the computational domain. The dimensions of the domain before scaling with the reference factors are *R* = 7 mm and *H* = 45 mm, with an average element size of 0.1 mm. The coupled set of PDEs (3.9) and (3.10), and (3.19) and (3.20) are discretized with the help of the FEM [26]. The fields *T*, *S*, *ψ* and *ω* are approximated as follows:

4.1 $$X(r,z,t)=\sum_{i}^{N}\alpha_{i}(r,z)\cdot u_{i}^{\left(X\right)}(t),$$

where X∈{T,S,ψ,ω}, *N* is the total number of degrees of freedom (d.f.) in the domain, *α*_*i*_ are the basis functions, in this case, first-order polynomials, and $u_{i}^{\left(X\right)}\in \{u^{\left(T\right)},u^{\left(S\right)},u^{\left(\psi \right)},u^{\left(\omega \right)}\}$ are the d.f. of the temperature, salinity, stream function and vorticity, respectively. Introducing the approximation (4.1) in the governing equations (3.9) and (3.10), and (3.19) and (3.20), multiplying with test functions and integrating by parts [34], and taking into account the boundary conditions from §3.3, leads to the following discrete systems of equations:

4.2 $$M^{\left(T\right)}{\dot{u}}^{\left(T\right)}+K^{\left(T\right)}u^{\left(T\right)}=0,$$

4.3 $$M^{\left(S\right)}{\dot{u}}^{\left(S\right)}+K^{\left(S\right)}u^{\left(S\right)}=0$$

4.4 $$\mathrm{and}\;\left[\begin{array}{cc} A & B\\ B^{t} & -C \end{array}\right]\left[\begin{array}{c} u^{\left(\omega \right)}\\ u^{\left(\psi \right)} \end{array}\right]=\left[\begin{array}{c} 0\\ F \end{array}\right],$$

where the individual entries of the matrices and vectors are given as

4.5 $$M_{i,j}^{\left(T\right)}=\int_{\Omega^{\prime }}r\left(1+\frac{1}{Ste}\delta^{\epsilon }(T-T_{f})\right)\alpha_{i}\alpha_{j}\;dr\;dz,$$

4.6 $$M_{i,j}^{\left(S\right)}=\int_{\Omega^{\prime }}r\alpha_{i}\alpha_{j}\;dr\;dz,$$

4.7 $$K_{i,j}^{\left(T\right)}=\int_{\Omega^{\prime }}\left[r\left(1+\frac{1}{Ste}\delta^{\epsilon }(T-T_{f})\right)\alpha_{i}V\cdot \nabla^{\prime }\alpha_{j}+\frac{r}{Pe_{T}}\nabla^{\prime }\alpha_{i}\cdot \nabla^{\prime }\alpha_{j}\right]\;dr\;dz,$$

4.8 $$K_{i,j}^{\left(S\right)}=\int_{\Omega^{\prime }}\left[r\alpha_{i}V\cdot \nabla^{\prime }\alpha_{j}+\frac{r}{Pe_{S}}\nabla^{\prime }\alpha_{i}\cdot \nabla^{\prime }\alpha_{j}\right]\;dr\;dz,$$

4.9 $$A_{i,j}=\int_{\Omega^{\prime }}\alpha_{i}\alpha_{j}\;dr\;dz,$$

4.10 $$B_{i,j}=\int_{\Omega^{\prime }}\left[\nabla^{\prime }\alpha_{i}\cdot \nabla^{\prime }\alpha_{j}+\alpha_{i}\frac{\hat{r}}{r}\cdot \nabla^{\prime }\alpha_{j}\right]\;dr\;dz,$$

4.11 $$C_{i,j}=\int_{\Omega^{\prime }}\left[\frac{1}{Da}\nabla^{\prime }\alpha_{j}\cdot \nabla^{\prime }\alpha_{i}+\frac{1}{Da}\frac{\hat{r}}{r}\cdot \nabla^{\prime }\alpha_{j}\alpha_{i}\right]\;dr\;dz$$

4.12 $$\mathrm{and}\;F_{i}=\int_{\Omega^{\prime }}\frac{r}{Re}\frac{\partial Ar}{\partial r}\alpha_{i}\;dr\;dz.$$

In the above equations, *α*_*i*_ denotes the Lagrangian basis function associated with the *i*th d.f. Since we only impose Dirichlet and zero Neumann boundary conditions, there are no boundary terms contributions to the right-hand side of equations (4.2)–(4.4). The integrals, given by (4.5)–(4.12), are evaluated numerically using Gaussian quadrature rules. The quadratures integrate exactly the resulting polynomial approximations.

Tracking the interface between solid and fluid requires the utilization of immersed techniques or re-meshing the model at every step. We avoid these complexities in the implementation by employing the following smoothed-step function:

4.13 $$\Theta (T-T_{f})=\frac{1}{2}\left(1+\tanh\left(\frac{5}{\Delta T}(T-T_{f})\right)\right),$$

where *T*_*f*_ is the fusion temperature of the fluid and Δ*T* is a free small parameter. The function will approximate a unit step function when Δ*T* is small.

With the help of equation (4.13), we construct the phase-dependent parameters such as the Péclet numbers and model both the Dirac delta function and the Darcy number as follows:

4.14 $$\delta^{\epsilon }(T-T_{f})=\frac{\nabla^{\prime }(T-T_{f})\cdot \nabla^{\prime }\Theta (T-T_{f})}{\|\nabla^{\prime }(T-T_{f}){\|}^{2}+\epsilon }$$

and

4.15 $$\frac{1}{Da}=\epsilon +\frac{{\left(1-\Theta (T-T_{f})\right)}^{2}}{\Theta {\left(T-T_{f}\right)}^{3}+\epsilon },$$

where ϵ is a small parameter introduced to avoid numerical instabilities. Equation (4.14) is obtained using the fact that the Dirac delta function is the derivative of the jump function, while equation (4.15) is inspired by the work of Carman in porous flow [35]. The regularized delta function $\delta^{\epsilon }$ as well as the matrices ***M***^(*T*)^ and ***K***^(*T*)^ depend on the current solution, i.e. the temperature, salinity, vorticity and the stream function, which is taken from the last converged solution during the time integration process.

To solve the system of ordinary differential equations (ODE) (4.2) and (4.3), we use an implicit Runge–Kutta method of fourth-order provided by the SUNDIALS library [36,37]. In particular, we employ the ARKODE time integrator [38], which allows us to use an adaptive time stepping with third-order embedding. The nonlinear matrices are updated at every time step. However, they are kept constant inside the Runge–Kutta iterations. Every time step, we solve the system (4.4) by using the LU factorization method implemented in the SuperLU_DIST library [39–41]. Inside the ODE integration iteration, it is required to solve two types of linear systems: ***MX*** = ***B*** and (***M*** + *γ****K***)***X*** = ***B*** where $M\in \{M^{\left(T\right)},M^{\left(S\right)}\}$, $K\in \{K^{\left(T\right)},K^{\left(S\right)}\}$ and *γ* is a scaled time step size. As ***M***^(*T*)^, ***M***^(*S*)^ are mass matrices with positive weight functions, they are symmetric and positive definite. Thus for the first type of system, we use the conjugate gradient method preconditioned with an algebraic multi-grid method. For the second problem, the system loses the symmetry owing to the convection terms in ***K***^(*T*)^, ***K***^(*S*)^, and we employ the generalized minimal residual method preconditioned with an algebraic multi-grid method. All linear iterative solvers are provided by the HYPRE library [42] within the MFEM library [29].

## 5. Results

Using the FEM discretization discussed in §4, we performed simulations with a set of parameters inspired by the work of Martin [7]. We set the radius and height of the container at 7 mm and 45 mm, respectively, while the inflow radius is 2 mm. Also, we set the initial values of the seawater at a temperature of $-2^{\circ }C$ and salinity of 3.5% wt. The injected brine is set at 22.5% wt salinity while its temperature is varied between $-10^{\circ }C$, $-15^{\circ }C$, $-16^{\circ }C$ and $-18^{\circ }C$. Moreover, the inflow rates are set from 100 mm^3^ s^−1^ to 500 mm^3^ s^−1^ in steps of 100 mm^3^ s^−1^, resulting in Reynolds numbers $Re=Q/2\pi R_{\mathrm{in}}\nu$ varying from Re≈2.27 to Re≈11.35. We also use the numerical values of the physical properties explained in appendix A. All of the simulations ran for 70 s. The link to a compilation of animations for each simulation can be found in appendix B.

**Figure 4.**
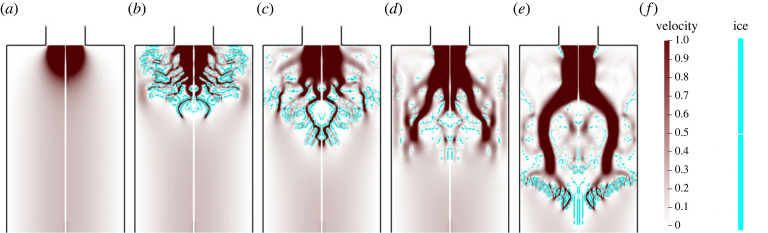
Time sequence of a simulation with an inflow rate 300 mm^3^ s^−1^ and inflow temperature $-10^{\circ }C$ at times: (*a*) 0.0 s, (*b*) 0.6 s, (*c*) 1.2 s, (*d*) 1.8 s and (*e*) 2.4 s. The velocity magnitude field (red and white) and the ice structure (cyan) are shown in each image, with the magnitude scales on the right side of the figure. (*f*) Scale.

As the formation of a brinicle is an evolutionary process, first, we analyse the beginning of the simulations. In figure 4, the first 3 s of a run with inflow rate 300 mm^3^ s^−1^ and inflow temperature $-10^{\circ }C$ are shown. The main feature of these figures is the ice branching structure shown in figure 4*b*. These structures are part of the phenomenon and can be seen clearly in a video from the British Broadcasting Corporation [4] where the camera operator provides a close-up of the tip of the brinicle. Thus, the proposed simulation procedure reproduces closely this phenomenon. Furthermore, analysing figure 4*c* closely we can see that the high-velocity regions correspond to the inner part of the narrow ice channels while the velocity at the ice walls is zero, demonstrating that the Brinkman term successfully models the lack of permeability of the ice structures. After some period of time, the brine breaks through the ice (figure 4*d*), and another dendrite structure can appear in the simulations (figure 4*e*). When the brine breaks through the last dendrite structure, the characteristic ice channel of the brinicle forms.

There is another important aspect regarding the symmetry boundary (*r* = 0). The ice fragments tend to attach to the boundary, as shown in figure 4*c*, producing not physically expected obstacles to the flow. This phenomenon is a result of the axisymmetric assumption made at the beginning of the analysis. The imposed Neuman conditions imply that local maxima or minima, along the normal to the boundary, could appear in the region, leading to this unexpected behaviour. A straightforward way to address this limitation is to eliminate the symmetry boundary with a three-dimensional cartesian formulation, although at the cost of a significantly more expensive simulation. Nevertheless, to approach this with our current model, we increased the diffusivity rate of the salt in the liquid phase by one order of magnitude, as shown in appendix A. Thus, it is easier for the brine flux to melt the ice near the boundary by balancing the salt concentration, hence removing the unexpected effect.

**Figure 5.**
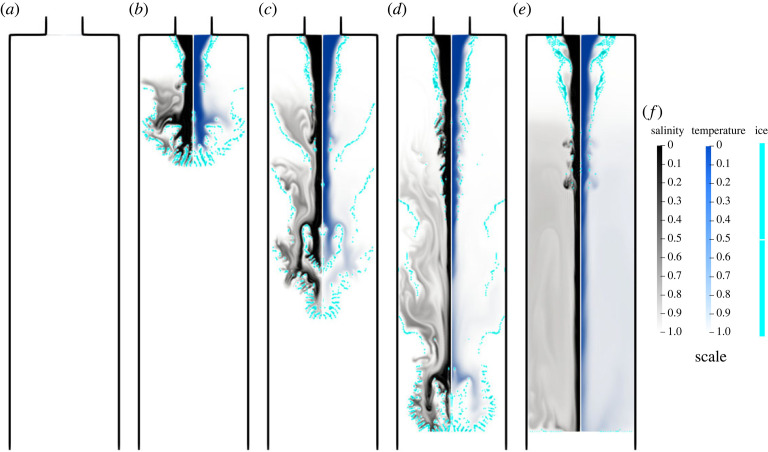
Sequence from simulation of an inflow rate 100 mm^3^ s^−1^ and inflow temperature $-10^{\circ }C$ for: (*a*) 0 s, (*b*) 3 s, (*c*) 6 s, (*d*) 9 s and (*e*) 70 s. In each image, the salinity field (black and white), the temperature field (blue and white) and the ice structure (cyan) are shown, with their corresponding scales at the right side of the figure. (*f*) Scale.

Figure 5 demonstrates the behaviour of a simulation with inflow rate 100 mm^3^ s^−1^ and inflow temperature $-10^{\circ }C$. The latter portrays that the evolution of the ice structure stabilizes around the brine channel resembling the cross-section of a brinicle in the sense of a hollow stalactite. It is important to denote that the ice tends to attach itself to the closed boundaries, which are the insulated ones according to §3.3. Hence, these act as nucleation points for the phase change. The latter is the principal mechanism from which thick ice structures appear. However, it has additional unexpected side effects. For example, some layers of ice appear in the middle of the channel attached to the lateral boundaries, preventing brine from flowing freely, as can be seen in figure 5*c*. Since the outflow boundary has the same conditions on the salinity and temperature as the closed boundary, ice also tends to appear near that part of the domain. This effect is shown in figure 5*e*, as a thin layer of ice at the bottom causes the brine to accumulate on the domain. Hence, the brine excess prevents further growth of the final ice structure.

**Figure 6.**
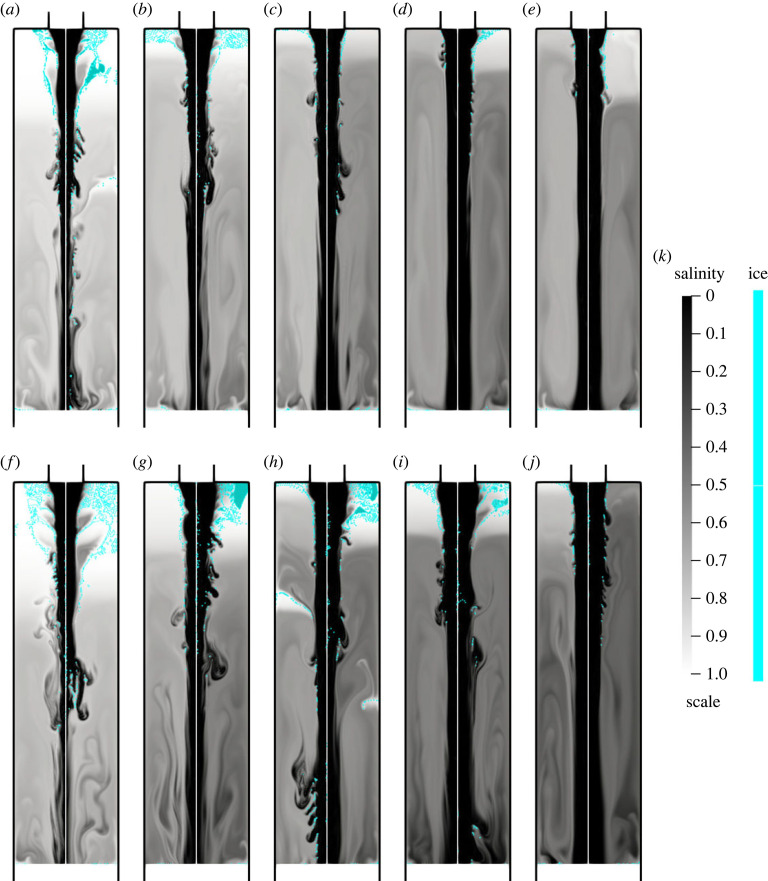
Comparison at time *t* = 60 s of the salinity (black and white) and the ice structure (cyan) between different inflow rates and temperatures. Each image corresponds to an inflow rate and two different inflow temperatures. (*a*) 100 mm^3^ s^−1^ $-10^{\circ }C\;(\mathrm{left})-15^{\circ }C\;(\mathrm{right})$, (*b*) 200 mm^3^ s^−1^ $-10^{\circ }C\;(\mathrm{left})-15^{\circ }C\;(\mathrm{right})$, (*c*) 300 mm^3^ s^−1^ $-10^{\circ }C\;(\mathrm{left})-15^{\circ }C\;(\mathrm{right})$, (*d*) 400 mm^3^ s^−1^ $-10^{\circ }C\;(\mathrm{left})-15^{\circ }C\;(\mathrm{right})$, (*e*) 500 mm^3^ s^−1^ $-10^{\circ }C\;(\mathrm{left})-15^{\circ }C\;(\mathrm{right})$, (*f*) 100 mm^3^ s^−1^ $-16^{\circ }C\;(\mathrm{left})-18^{\circ }C\;(\mathrm{right})$, (*g*) 200 mm^3^ s^−1^ $-16^{\circ }C\;(\mathrm{left})-18^{\circ }C\;(\mathrm{right})$, (*h*) 300 mm^3^ s^−1^ $-16^{\circ }C\;(\mathrm{left})-18^{\circ }C\;(\mathrm{right})$, (*i*) 400 mm^3^ s^−1^ $-16^{\circ }C\;(\mathrm{left})-18^{\circ }C\;(\mathrm{right})$, (*j*) 500 mm^3^ s^−1^ $-16^{\circ }C\;(\mathrm{left})-18^{\circ }C\;(\mathrm{right})$. (*k*) Scale.

In figure 6, we show the comparison between all the test cases for a time period of 1 min. It can be seen on these figures that ice walls appear for all inflow temperatures with inflow rates of 100 mm^3^ s^−1^ and 200 mm^3^ s^−1^, while for 300 mm^3^ s^−1^ and 400 mm^3^ s^−1^, the ice layer only forms at low temperatures ($-16{\;}^{\circ }C$ and $-18{\;}^{\circ }C$). Note that with an inflow of 500 mm^3^ s^−1^, the ice is too thin to form the expected structures. Therefore, the lower the inflow rate and temperature, the better the chance to form structures similar to a brinicle. The latter corresponds closely to the experimental observations in [7] where colder inflow temperatures tend to produce better-defined brinicle structures. However, brinicles with high inflow rates such as 500 mm^3^ s^−1^ have been observed in [7]. We can relate this discrepancy to the brine accumulation because of the freezing of the outflow boundary since this effect is more pronounced for higher inflow rates, as can be seen in figure 6. The ice formation is damped owing to this effect. One way to avoid it is to increase the mesh size, which decreases the salt accumulation effect but raises the computational cost. Another approach would be to consider a different type of boundary condition for the outflow such as a Robin condition. The above possibility together with a full three-dimensional model of the process deserves further detailed study in the future.

## 6. Conclusion

We constructed a general theoretical model that accounts for phase change phenomena associated with heat and mass transfer throughout the domain for simulating the evolution of brinicles. The proposed model is highly nonlinear with many constituents at play. The numerical solution is obtained with the help of the FEM. The used Brinkman penalization removed the need for re-meshing or for the employment of immersed techniques, and we were able to capture the phase change geometry. A smoothed Dirac delta term can account for the latent heat, which is a crucial feature of the model, as shown in figures 6 and 5. The proposed methodology is able to generate structures with the shape of a brinicle. Furthermore, as shown in figure 4, the model reproduced the dendrite structure that forms on the brinicle [4]. We also found that with inflow rates of 100 mm^3^ s^−1^ and 200 mm^3^ s^−1^, the brinicle growth is captured in all cases. However, for faster flows of 300 mm^3^ s^−1^ and 400 mm^3^ s^−1^ it is only formed at low inflow temperatures ($-16^{\circ }C$ and $-18^{\circ }C$). The latter suggests that for lower temperatures, the structure should appear with a higher probability as observed experimentally in [7]. Our model was unable to recreate brinicles with high inflows. The reason behind such behaviour is the brine accumulation caused by the freezing of the outflow boundary. The brine accumulation and the ice that forms near the symmetry and insulated boundaries are a consequence of the boundary conditions. Imposing Neumann conditions means that the field value on the boundary must be local maxima or minima, which can result in numerical artefacts and the unexpected behaviour discussed in §5. These conditions result from the assumption that the insulating boundaries are perfect insulators and the outflow is far from the inlet. Therefore, additional research is necessary to find more appropriate conditions that approximate better the experimental behaviour. Another source of numerical artefacts is the symmetry boundary. We resolved these problems with indirect methods like increasing the diffusivity rate. However, a full three-dimensional simulation must be considered to avoid adding an artificial boundary.

Aside from the boundaries, the model can be significantly improved by removing the assumption for creeping flow and replacing the Stokes model with the full Navier–Stokes equations. The current fluid flow model is selected mainly owing to its low computational cost. However, the full Navier–Stokes equations represent better the physical behaviour for the considered cases with Reynolds numbers varying from Re≈2.27 to Re≈11.35. The resulting flow regime is laminar with relatively small convective effects. Improving the model will mainly impact the initial stages of the brinicle formation, i.e. during the branching process, the flow changes direction, and the inertial effects will lead to differences in the velocity distribution of the fluid. On the other hand, once an ice structure is stabilized around the brine channel, the flow will be similar to a pipe flow, also known as Poiseuille flow, and the difference between the Stokes and the Navier–Stokes models is negligible. Other functional forms for the parameters shown in appendix A could be considered as some of the constant properties might also depend on salinity and temperature. Consequently, there is substantial work to be done in the numerical simulation of these phase-changing systems that we hope can be studied more thoroughly in the future.

Finally, it is important to note that this approach represents an advance in modelling these types of complex systems since it shows, to our knowledge, for the first time that the mentioned fully coupled system of equations can reproduce many aspects of the physical evolution of brinicle formation. Hence we believe our contribution will be useful to the simulation of ice structures which are important in climate studies [20] and to similar phenomena in the industry, like the modelling of phase-changing materials [10].

## Data Availability

Data and relevant code for this research work are stored in GitHub: https://github.com/fegomezl/Brinicle and have been archived within the Zenodo repository: https://www.doi.org/10.5281/zenodo.8384885 [43]. Supplementary material is available online [44].

## Appendix A. Physical parameters

The appendix describes in detail the physical properties of the model and the dimensionless numbers used in the scaling process explained in §2. First, we discuss the fusion temperature. The fusion temperature of the seawater is a function of salinity that, according to Hall *et al.* [45], can be represented as

A1 $$\begin{array}{rl} & T_{f}\;(S)=aS+bS^{3},\\ & a=-0.6037\\ \mathrm{and}\; & b=-0.00058123, \end{array}\}$$

where *T*_*f*_ is in degrees Celsius (${\;}^{\circ }C$) and *S* in weight percentage (%wt). The dimensionless fusion temperature is found by applying the linear map (2.1) to both *T*_*f*_ and *S*.

From equations (3.9) and (3.10), we derive the following dimensionless parameters:

— *Péclet number:* *Pe* = 2*πR*_in_*α*/*Q*—ratio between diffusive and convective rates; and
— *Stefan number:* *Ste* = (*c*_mean_|*T*_in_ − *T*_0_|)/*L*—ratio between sensible and latent heat.

In the above, we introduce the latent heat *L*, the mean specific heat capacity *c*_mean_ between both phases and the diffusivity constant *α* which refers to the salinity and temperature equations in the liquid and solid phases.

A close inspection of equations (3.19) and (3.20) reveals the next set of dimensionless numbers:

— *Reynolds number:* *Re* = *Q*/2*πR*_in_*ν*—ratio between viscous and inertial forces;
— *Archimedes number:* $Ar=gR_{\mathrm{in}}^{3}\rho /\nu^{2}$—ratio between viscous and buoyant forces; and
— *Darcy number:* $Da=R_{\mathrm{in}}^{2}/\eta$—permeability penalization number.

The Reynolds number is constant since the viscosity *ν* and the other equation parameters are assumed time-independent. The construction of the Darcy number follows equation (4.15). While the Archimedes number depends on the gravity *g* and other constant parameters, it is also linked with the relative density *ρ* which is a function of both temperature and salinity. Even if density variations between phases are neglected, as they are too complex to model, we do account for these changes in the density inside the liquid phase to produce buoyant forces. We evaluate the relative density of the liquid phase as a function of concentration and temperature with the equation of state for seawater proposed by Millero & Huang [46]:

A2 $$\begin{array}{rl} & \rho (T,S)=A(T)S+B(T)S^{1.5}+C(T)S^{2},\\ & A(T)=a_{0}+a_{1}T+a_{2}T^{2}+a_{3}T^{3}+a_{4}T^{4},\\ & B(T)=b_{0}+b_{1}T+b_{2}T^{2}\\ \mathrm{and}\; & C(T)=c_{0}, \end{array}\}$$

where *T* is given in degrees Celsius (${\;}^{\circ }C$), *S* in weight percentage (%wt) and *a*_*i*_, *b*_*j*_, *c*_*k*_ are constants that can be seen in table 1.

**Table 1.** Empirical constants of seawater equation of state from equation (A 2).

number	*a*	*b*	*c*
0	8.25 × 10^−3^	−1.83 × 10^−4^	4.89 × 10^−5^
1	−4.11 × 10^−5^	3.33 × 10^−6^	
2	7.73 × 10^−7^	−5.60 × 10^−8^	
3	−8.32 × 10^−9^		
4	5.52 × 10^−11^		

Finally, table 2 contains the values of the remaining constants of the system.

**Table 2.** Summary of numerical values for diffusivity rates *α* of temperature *T* and salinity *S* in each phase liquid *l* or solid *s*, mean heat capacity between phases *c*_mean_, latent heat *L*, kinematic viscosity *ν* and gravitational acceleration *g*. (All of the values were taken from Martin [7]. For the liquid salinity diffusivity, a bigger order is considered because of numerical instabilities.)

$\alpha_{l}^{T}\;({\mathrm{mm}}^{2}\;s^{-1})$	$\alpha_{s}^{T}\;({\mathrm{mm}}^{2}\;s^{-1})$	$\alpha_{l}^{S}\;({\mathrm{mm}}^{2}\;s^{-1})$	$\alpha_{s}^{S}\;({\mathrm{mm}}^{2}\;s^{-1})$	$c_{\mathrm{mean}}\;(\mathrm{kJ}/({\mathrm{kg}}^{\circ }C))$	*L* (kJ kg^−1^)	*ν* (mm^2^ s^−1^)	*g* (mm^2^ s^−1^)
0.14	1.13	0.01	0	2.94	322.7	6.8	9800

## Appendix B. Electronic supplementary material

The animations corresponding to the simulations shown in figures 4, 5 and 6 are available at https://github.com/fegomezl/Brinicle. Please also see the electronic supplementary material.
